# Generalized Models for Rock Joint Surface Shapes

**DOI:** 10.1155/2014/171873

**Published:** 2014-07-24

**Authors:** Shigui Du, Yunjin Hu, Xiaofei Hu

**Affiliations:** ^1^College of Civil Engineering, Shaoxing University, Shaoxing 312000, China; ^2^College of Civil Engineering and Architecture, Zhejiang University, Hangzhou 310058, China; ^3^Zhejiang Industry Polytechnic College, Shaoxing 312000, China

## Abstract

Generalized models of joint surface shapes are the foundation for mechanism studies on the mechanical effects of rock joint surface shapes. Based on extensive field investigations of rock joint surface shapes, generalized models for three level shapes named macroscopic outline, surface undulating shape, and microcosmic roughness were established through statistical analyses of 20,078 rock joint surface profiles. The relative amplitude of profile curves was used as a borderline for the division of different level shapes. The study results show that the macroscopic outline has three basic features such as planar, arc-shaped, and stepped; the surface undulating shape has three basic features such as planar, undulating, and stepped; and the microcosmic roughness has two basic features such as smooth and rough.

## 1. Introduction

The morphology of rock joint surface is being more and more frequently discussed by authors specializing in various aspects of geology and rock mechanics [1, 2]. Generalized models of surface shapes are the foundation for mechanism studies on the mechanical effects of rock joint surface shapes. Establishment of the generalized model for the joint surface shapes can help to understand the physical meanings of JRC (joint roughness coefficient) and discuss the friction and abrasion mechanisms of the JRC-JCS (joint compressive strength) model [3–6]. It is an effective way for perfecting the empirical estimation method of joint shear strength and making the empirical estimation method practical to carry out shear strength tests, numerical simulation, comparison analyses, and engineering application study on joints with different generalized model of surface shape.

**Figure 1:**
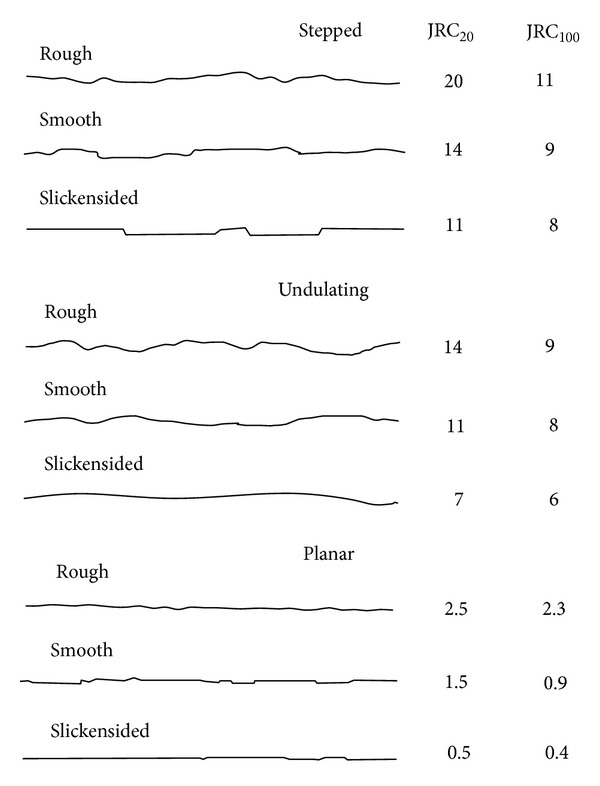
Typical roughness profiles and corresponding range of JRC [7].

According to the recommendation of ISRM (International Society of Rock Mechanics), the surface shape of joint could be described by a waviness and by an unevenness [9, 10]. The waviness describes large scale undulations which, if interlocked and in contact, cause dilation during shear displacement since they are too large to be sheared off. The unevenness describes small scale roughness that tends to be damaged during shear displacement unless the joint walls are of high strength and/or the stress levels are low, so that dilation can also occur on these small scale features. The classification models suggested by the ISRM are presented in Figure 1. From the figure, it can be seen that the waviness can be classified into stepped, undulating, and planar; and the unevenness can be classified into rough, smooth, and slickensided.

**Figure 2:**
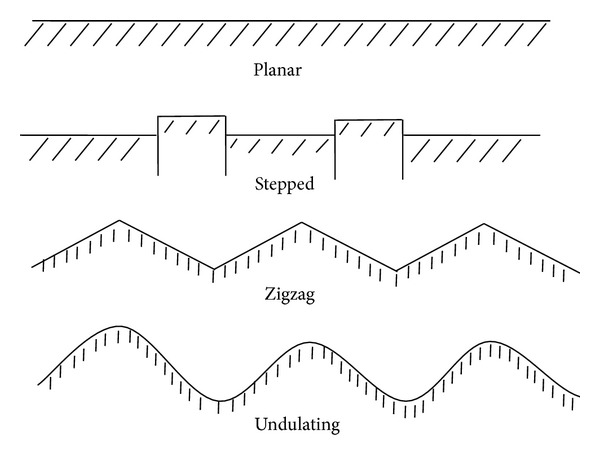
Type of joint undulating shape [8].

Sun [8] used an undulation and a roughness to describe the surface shape of rock joint. Effect of surface undulation on mechanical characteristics of rock joint has two aspects. One is the undulation difference, the other is the undulation angle. The undulation can be classified into planar, stepped, zigzag, and undulating (see Figure 2). Effect of surface roughness on mechanical characteristics of rock joint is included in the test results of joint sample. The roughness can be classified into rough, smooth, and mirror-like.

According to the aforesaid analyses, it can be seen that the waviness and unevenness recommended by ISRM are equivalent to the undulation and roughness suggested by Sun [8], respectively. Although they classified the joint surface shape into two levels, they did not propose the borderline for the division of the two level shapes, which results in the inconvenience for application. In this paper, on the basis of extensive field investigations of rock joint surface shapes, generalized models for three level shapes such as macroscopic outline, surface undulating shape, and microcosmic roughness were established through statistical analyses of 20,078 rock joint surface profiles. The relative amplitude of profile curves was used as a borderline for the division of different level shapes.

## 2. Essential Factors of Joint Surface Shape

**Figure 3:**
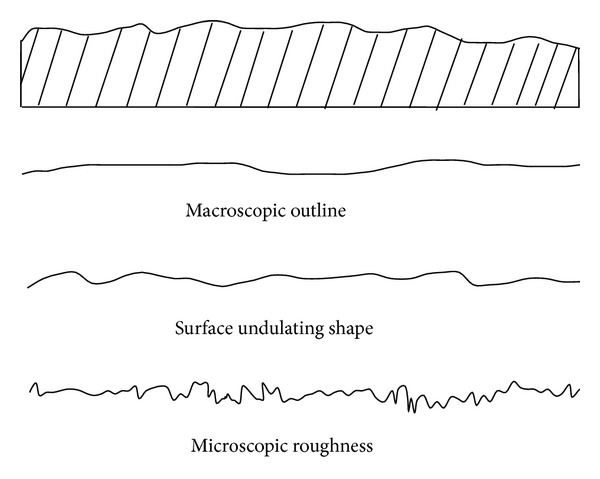
Essential factors of joint surface shape.

In order to ease the description and study of complex joint surface shapes, the joint surface shape should be classified firstly. Then, classification description of the joint surface shape can be carried out. Essential factors of joint surface shape are the basis of classification description. Study results of Du [11] show that the joint surface shape consists of three essential factors, named macroscopic outline, surface undulating shape, and microcosmic roughness (see Figure 3).

### 2.1. Macroscopic Outline

The macroscopic outline is the maximum level of geometric profile of joint surface and reflects the entire geometric shape of joint surface. The macroscopic outline is represented by the envelope curve of the apices or vales of surface undulating shape. The macroscopic outline is relevant to rock formation, characteristics of stress propagation in rock masses, and expansion and perforation mode of joints. For example, tensile joint surfaces formed by the perforation of several panache structures made by mode I crack expansion are usually arc-shaped; and shear joint surfaces formed by mode II crack expansion are commonly planar.

### 2.2. Surface Undulating Shape

The surface undulating shape constitutes undulating profile of apices and vales on the joint surface and reflects the undulating degree of joint surfaces. The surface undulating degree is often represented by the undulation amplitude, the relative amplitude, and the undulation angle. It is the dominant factor affecting the mechanical properties of joints.

The surface undulating shape is the exhibition of joint wall rock formation and size, shape, and distribution of coarse grains (mineral aggregation, gravel, and nodule) on the joint surface. It is also the records of the stress distribution and propagation mode in rock masses and crack expansion and perforation mode on the joint surface during the process of joint formation. For homogeneous rock, tensile joint surface formed by the perforation of panache structures (consists of pinnated veins, ridged sign, and steep bank) made by mode I crack expansion is usually more undulant; shear joint surface formed by mode II crack expansion consists of slickensided and stepped features and is commonly less undulant; and undulating degree of joint surfaces formed by mode III crack expansion is intervenient between those of the abovementioned two kinds of joint surfaces. With respect to rock containing nodule, gravel, or coarse mineral aggregation, the joint surface formed by through-grain crack is less undulant, and the joint surface formed by along-grain crack is more undulant.

### 2.3. Microcosmic Roughness

The microcosmic roughness is the minimum level of undulating shape of joint surface and reflects the sublevel tiny geometric character of slope surface on the apices and vales. It is the exhibition of distribution and arrangement character of mineral grains or tiny mineral aggregations on the joint surface.

The stress distribution in rock masses and the crack expansion mode of joints have no significant effect on the microcosmic roughness. However, ingredient of mineral or crystal, rock formation, size and shape of mineral crystal, arrangement of mineral, and its outcrop status on the joint surface determine the basic properties of microcosmic roughness.

## 3. Typical Surface Profile Curves of Rock Joints

**Figure 4:**
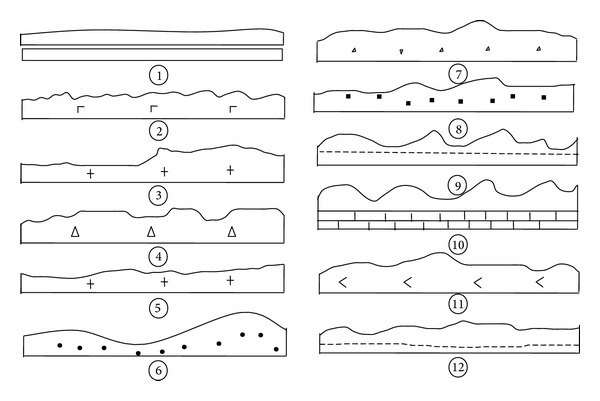
Typical profile curves of joint surface (sample size is 10 cm and scale is 1 : 2). ① Planar and smooth (carbonaceous slate foliation). ② Planar and rough (basaltic columnar joint). ③ Single stepped (coarse-grained granite joint). ④ Multistepped (volcanic breccia joint). ⑤ Slow undulating (fine-grained granite joint). ⑥ Undulating (siltstone ripple bedding). ⑦ Undulating (breccia-bearing fusion tuff joint). ⑧ Undulating (fine-sandstone joint). ⑨ Compound undulating (calcareous clay rock joint). ⑩ Compound undulating (calcilutite joint). ⑪ Planar-undulating (mixed hornblendite joint). ⑫ Stepped-undulating (calcareous clay rock joint).

**Figure 5:**
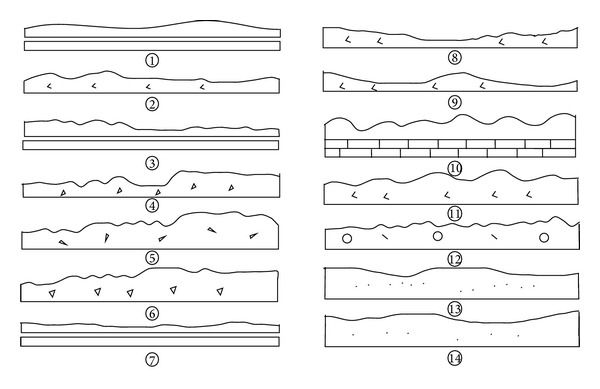
Typical profile curves of joint surface (sample size is 15 cm and scale is 1 : 3). ① Planar and smooth (carbonaceous slate foliation). ② Planar and rough (plagioclase hornblendite joint). ③ Single stepped (phyllite joint). ④ Compound single stepped (volcanic breccia joint). ⑤ Multistepped (volcanic breccia joint). ⑥ Compound multistepped (volcanic breccia joint). ⑦ Undulating (carbonaceous slate foliation). ⑧ Undulating (plagioclase hornblendite joint). ⑨ Undulating (plagioclase hornblendite joint). ⑩ Undulating (calcilutite joint). ⑪ Compound undulating (mixed hornblendite joint). ⑫ Asymmetric undulating (breccia-bearing fusion tuff joint). ⑬ Asymmetric undulating (siltstone ripple bedding). ⑭ Symmetric undulating (siltstone ripple bedding).

**Figure 6:**
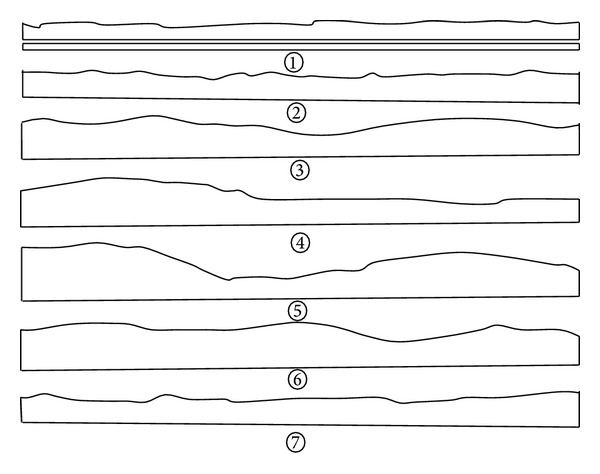
Typical profile curves of joint surface (sample size is 22 cm and scale is 1 : 3). ① Planar and smooth (carbonaceous slate foliation). ② Planar and rough (coarse-grained granite joint). ③ Undulating (siltstone ripple bedding). ④ Stepped-undulating (siltstone ripple bedding). ⑤ Stepped-undulating (siltstone ripple bedding). ⑥ Stepped-undulating (siltstone ripple bedding). ⑦ Planar-undulating (plagioclase hornblendite joint).

Great deals of field investigations of rock joint surface shapes have been done by the authors since 1992. Twenty thousand and seventy eight profile curves have been recorded by use of the profilograph instrument [12]. These curves belong to six categories of hard joint surfaces (such as stratification, foliation, phyllite, schistosity, fault, and joint) with various sample lengths (such as 10 cm, 15 cm, 22 cm, 30 cm, 40 cm, 50 cm, 60 cm, 70 cm, 80 cm, 90 cm, and 100 cm). These joint surfaces were sampled from different kinds of rock masses in different regions of China [13–15]. Studies of generalized models of joint surface shapes were mainly based on the typical profile curves of joint surfaces with three different sample lengths of 10 cm (see Figure 4), 15 cm (see Figure 5), and 22 cm (see Figure 6).

## 4. Generalized Models of Rock Joint Surface Shapes

Based on the qualitative descriptions and statistical analyses of surface shape parameters for the abovementioned typical profile curves, generalized models of rock joint surface shapes can be obtained.

### 4.1. Generalized Models of Macroscopic Outline

**Figure 7:**
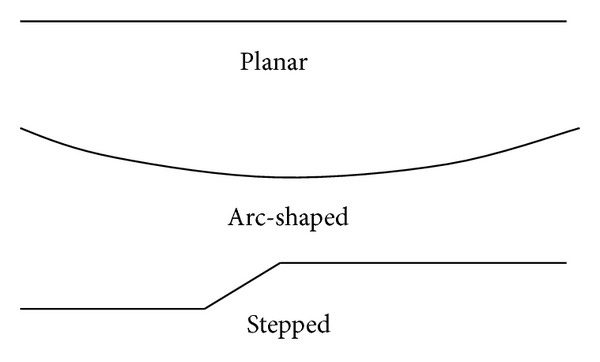
Generalized models of macroscopic outline.

The macroscopic outline represents the entire geometric shape of joint surface and can be classified by drawing the envelope curve of the apices or vales of the surface undulating shape. The field investigations show that the macroscopic outline can be classified as follows (see Figure 7).

1. Planar: the macroscopic outline of shear joint caused by shear stress or incomplete joint formed by interdissected is commonly planar. The macroscopic outline of marine sedimentary rock bedding and foliation, phyllite, and schistosity formed by metamorphism is also planar mainly.
2. Arc-shaped: the macroscopic outline of tensile joint caused by tension stress or continental sedimentary rock bedding is commonly arc-shaped.
3. Stepped: two or more planar joints communicated by minor implicit joints form stepped macroscopic outline.

### 4.2. Generalized Models of Surface Undulating Shape

**Figure 8:**
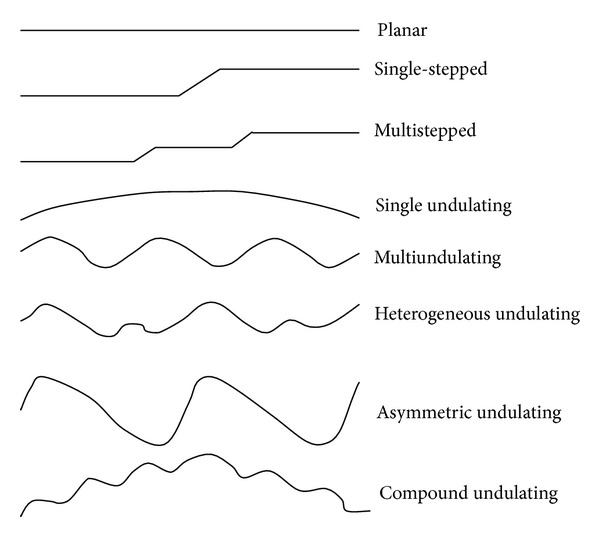
Generalized models of surface undulating shape.

The field investigations and statistical analyses show that the surface undulating shape has three basic features (planar, stepped, and undulating) and several compound features (such as planar-stepped, planar-undulating, and stepped-undulating, etc.) (see Figure 8).

1. Planar: if the surface undulating shape of familiar scale rock joint is planar, its relative amplitude *R*_*A*_ is always less than 1/200 or its JRC is less than 2 if sampling length is 10 cm. For shear joint caused by shear stress, when its wall is uniformly distributed, cryptomere or vitreous magmatic rock (such as basalt), fine-grained sedimentary rock (such as clay rock), or fine-grained metamorphic rock (such as slate) planar surface undulating shape may form. The statistical results show that the possibility of rock joint with planar surface undulating shape is rare if the sampling length is more than 15 cm.
2. Stepped: to rock joint with stepped surface undulating shape, its relative amplitude *R*_*A*_ is more than 1/200. The stepped joint is mostly caused by shear stress and its wall is homogeneous fine-grained rock. Due to friction and avulsion during the course of interslip of the two joint walls, stepped and/or antistepped features can be formed so its surface undulating shape is stepped. To tensile joint caused by tension stress, panache formation will be always formed on the joint surface and stepped surface undulating shape can be formed in the transition zone between two panache formations.
3. Undulating: the undulating is the most familiar surface undulating shape of rock joint and its relative amplitude *R*_*A*_ is more than 1/200. Whether shear joint or tensile joint, whether homogeneous wall rock or heterogeneous wall rock, and whether fine-grained wall rock or coarse-grained wall rock, all can form undulating surface shape. The experimental results show that fresh rock joint caused by tension rupture can form zigzag surface undulating shape [3, 8]. The zigzag surface undulating shape will be changed to the undulating surface shape due to the reconstruction of geological tectonic force. That is to say, the zigzag surface undulating shape cannot be reserved for natural rock joint.

In conclusion, the generalized model for surface undulating shape of rock joint is mainly undulating or planar-undulating and stepped-undulating. The undulating shape is the generalized model for the great majority of hard rock joints. Generally, if rock joint size is small, its surface undulating shape is mainly a single feature (planar, stepped, or undulating); the surface undulating shape will exhibit compound features with the increase of joint size (more than 20 cm).

### 4.3. Generalized Models of Microcosmic Roughness

**Figure 9:**
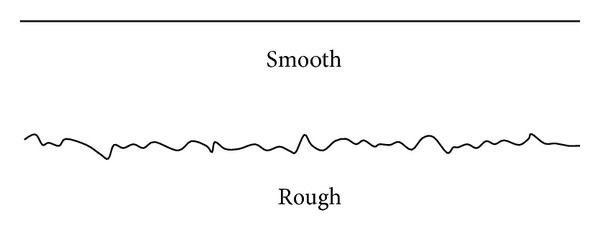
Generalized models of microcosmic roughness.

The field investigations and statistical analyses show that the microcosmic roughness can be classified into two basic features of smooth and rough (see Figure 9).

1. Smooth: for rock joint with smooth microcosmic roughness, its relative amplitude *R*_*A*_ is less than 1/600. Joint surfaces with wall rock made of uniformly distributed fine grains, like slate and clay rock, are often smooth.
2. Rough: to rock joint with rough microcosmic roughness, its relative amplitude *R*_*A*_ is more than 1/600 and less than 1/200. To joint with wall rock made of nonuniformly distributed fine grains or coarse grains or joint with discontinuous and nonuniform stress propagation during the course of formation, its microcosmic roughness is always rough.

## 5. Case Study for Generalized Models of Rock Joint Surface Shapes

**Figure 10:**
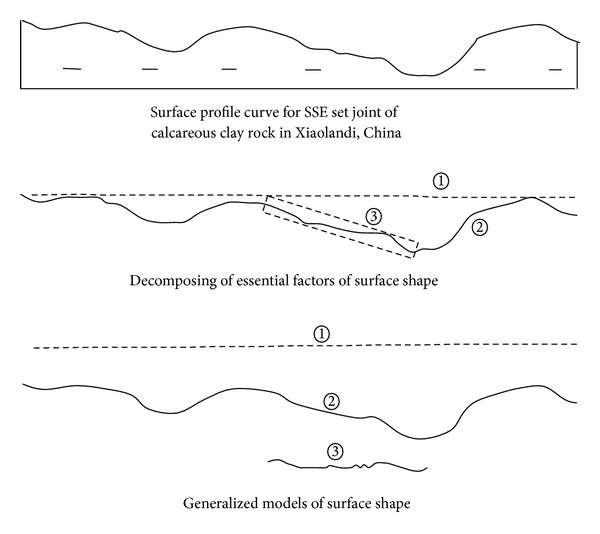
Case study of generalized models of rock joint surface (sample size is 10 cm). (1) Macroscopic outline (planar). (2) Surface undulating shape (undulating). (3) Microcosmic roughness (roughness).

The analysis procedure of generalized models of surface shapes for the profile curve of SSE set joint of calcareous clay rock in Xiaolangdi, China, is shown in Figure 10. According to the familiar scale undulating shape of profile curve, it can be seen that the surface undulating shape of the abovementioned joint is undulating (see Figure 10②). Drawing envelope curve for the undulating surface, it can be obtained that the macroscopic outline of the abovementioned joint is planar (see Figure 10①). Taking a wave surface from the undulating surface for analysis, it can be seen that the microcosmic roughness of the abovementioned joint is rough (see Figure 10③).

## 6. Conclusions

Based on amounts of field investigations of rock joint surface shapes, the generalized models for the three level shapes such as macroscopic outline, surface undulating shape, and microcosmic roughness were established through the statistical analyses of surface profile curves of rock joints. The relative amplitude of profile curves was used as a borderline for the division of different level shapes. Several conclusions can be obtained as follows.

1. The rock joint surface shape should be classified three different level shapes to describe objectively. Accordingly, the generalized models of the three different level surface shapes should be discussed, respectively.
2. The macroscopic outline has three basic features named planar, arc-shaped, and stepped. Shear joint caused by shear stress and incomplete joint formed by interdissected is commonly planar. Marine sedimentary rock bedding and foliation, phyllite, and schistosity formed by metamorphism are also planar mainly. Continental sedimentary rock bedding is commonly arc-shaped. However, the stepped macroscopic outline is the combined feature of the planar macroscopic outline.
3. The surface undulating shape has three basic features named planar, undulating, and stepped. The generalized model for surface undulating shape of rock joint is mainly undulating or planar-undulating and stepped-undulating. Generally, if the rock joint length is small, its surface undulating shape is mainly a single feature; the surface undulating shape will exhibit compound features with the increase of joint length.
4. The microcosmic roughness has two basic features named smooth and rough. Joint with wall rock made by uniformly distributed fine grains is often smooth. To joint with wall rock made by nonuniformly distributed fine grains or coarse grains or joint with discontinuous and nonuniform stress propagation during the course of formation, its microcosmic roughness is always rough.

## References

[1] MŁynarczuk M (2010). Description and classification of rock surfaces by means of laser profilometry and mathematical morphology. *International Journal of Rock Mechanics and Mining Sciences*.

[2] Cao P, Jia HQ, Liu TY, Pu C, Fan X (2011). Fractal analysis of three-dimensional topography characteristics of rock joint surface. *Chinese Journal of Rock Mechanics and Engineering*.

[3] Barton N (1973). Review of a new shear-strength criterion for rock joints. *Engineering Geology*.

[4] Barton N, Choubey V (1977). The shear strength of rock joints in theory and practice. *Rock Mechanics Felsmechanik Mécanique des Roches*.

[5] Yang Z, Lo SC, Di CC (2001). Reassessing the joint roughness coefficient (JRC) estimation using Z_2_. *Rock Mechanics and Rock Engineering*.

[6] Geertsema AJ (2002). The shear strength of planar joints in mudstone. *International Journal of Rock Mechanics and Mining Sciences*.

[7] Brown ET (1981). *Rock Characterization, Testing and Monitoring—ISRM Suggested Methods*.

[8] Sun GZ *Structural Mechanics of Rock Mass*.

[9] Zhao J (1997). Joint surface matching and shear strength. Part A: joint matching coefficient (JMC). *International Journal of Rock Mechanics and Mining Sciences & Geomechanics Abstracts*.

[10] Fardin N, Stephansson O, Jing L (2001). The scale dependence of rock joint surface roughness. *International Journal of Rock Mechanics and Mining Sciences*.

[11] Du SG (1999). *Engineering Properties of Rock Discontinuities*.

[12] Du S, Hu Y, Hu X (2009). Measurement of joint roughness coefficient by using profilograph and roughness ruler. *Journal of Earth Science*.

[13] Du SG (1994). The directive statistical evaluation of rock joint roughness coefficient. *Journal of Engineering Geology*.

[14] Du SG, Fang LB (1999). Statistical estimation for rock joint roughness coefficient. *Chinese Journal of Geophysics*.

[15] Du SG (2004). *Empirical Estimation for the Shear Strength of Rock Discontinuities*.

